# Use of Adjunctive Therapy in Acute Kawasaki Disease in Latin America

**DOI:** 10.3389/fped.2020.00442

**Published:** 2020-09-15

**Authors:** Brenda Fortuna-Reyna, Emelia V. Bainto, Rolando Ulloa-Gutierrez, Luis M. Garrido-García, Dora Estripeaut, Olguita del Águila, Virgen Gómez, Enrique Faugier-Fuentes, Greta Miño-León, Sandra Beltrán, Fernanda Cofré, Enrique Chacón-Cruz, Patricia Saltigeral-Simental, Lucila Martínez-Medina, Lourdes Dueñas, Kathia Luciani, Francisco J. Rodríguez-Quiroz, German Camacho-Moreno, Tamara Viviani, Martha I. Alvarez-Olmos, Heloisa Helena de Sousa Marques, Eduardo López-Medina, María C. Pirez, Adriana H. Tremoulet, The Kawasaki Disease REKAMLATINA Network Study Group

**Affiliations:** ^1^Universidad Autónoma de Nuevo León, Hospital Universitario “Dr. José Eleuterio González”, Monterrey, Mexico; ^2^University of California, San Diego, San Diego, CA, United States; ^3^California/Rady Children's Hospital San Diego, San Diego, CA, United States; ^4^Servicio de Infectología, Hospital Nacional de Niños “Dr. Carlos Sáenz Herrera”, San José, Costa Rica; ^5^Centro de Ciencias Médicas, Caja Costarricense de Seguro Social (CCSS), San José, Costa Rica; ^6^Servicio de Cardiología, Instituto Nacional de Pediatría, Ciudad de México, Mexico; ^7^Servicio de Infectología, Hospital del Niño Dr. José Renán Esquivel, Ciudad Panamá, Panama; ^8^Unidad de Infectología Pediátrica, Hospital Nacional Edgardo Rebagliati Martins, Lima, Peru; ^9^Servicio de Infectología, Centro Médico Universidad Central del Este Hospital y Hospital Infantil “Dr. Robert Reid Cabral”, Santo Domingo, Dominican Republic; ^10^Servicio de Reumatología, Hospital Infantil de México Federico Gómez, Ciudad de México, Mexico; ^11^Servicio de Infectología, Hospital del Niño “Francisco de Icaza Bustamante”, Guayaquil, Ecuador; ^12^Servicio de Infectología, Clínica Colsanitas, Bogotá, Colombia; ^13^Servicio de Infectología, Hospital Roberto del Río, Santiago, Chile; ^14^Servicio de Infectología, Hospital General de Tijuana, Tijuana, Mexico; ^15^Servicio de Infectología, Instituto Nacional de Pediatría y Hospital Infantil Privado, Ciudad de México, Mexico; ^16^Servicio de Infectología, Centenario Hospital Miguel Hidalgo, Aguascalientes, Mexico; ^17^Servicio de Infectología, Hospital de Niños Benjamín Bloom, San Salvador, El Salvador; ^18^Servicio de Infectología, Hospital de Especialidades Pediátricas Omar Torrijos Herrera, Caja de Seguro Social, Ciudad de Panamá, Panama; ^19^Servicio de Reumatología, Instituto Hondureño de Seguridad Social, Tegucigalpa, Honduras; ^20^Servicio de Infectología, Fundación HOMI Hospital Pediátrico de la Misericordia & Universidad Nacional de Colombia, Bogotá, Colombia; ^21^Servicio de Infectología, Hospital Sotero del Río, Santiago, Chile; ^22^Servicio de Infectología, Fundación Cardioinfantil & Universidad El Bosque, Bogotá, Colombia; ^23^Servicio de Infectología, Hospital Das Clinicas da Faculdade Medicina de la USP, São Paolo, Brazil; ^24^Centro de Estudios en Infectología Pediátrica, Departamento de Pediatría, Universidad del Valle y Centro Médico Imbanaco, Cali, Colombia; ^25^Servicio de Infectología, Hospital Pediátrico Centro Hospitalario Pereira Rossell, Montevideo, Uruguay

**Keywords:** Kawasaki disease, Latin America, steroids, infliximab, adjunctive therapy

## Abstract

**Objective:** To characterize the use of adjunctive therapy in Kawasaki disease (KD) in Latin America.

**Methods:** The study included 1,418 patients from the Latin American KD Network (REKAMLATINA) treated for KD between January 1, 2009, and May 31, 2017.

**Results:** Of these patients, 1,152 received only a single dose of IVIG, and 266 received additional treatment. Age at onset was similar in both groups (median 2 vs. 2.2 years, respectively). The majority of patients were male (58 vs. 63.9%) and were hospitalized with the first 10 days of fever (85.1 vs. 84.2%). The most common adjunctive therapy administered was steroids for IVIG-resistance, followed by additional doses of IVIG. The use of biologics such as infliximab was limited. KD patients who received adjunctive therapy were more likely to have a lower platelet count and albumin level as well as a higher Z score of the coronary arteries.

**Conclusion:** This is the first report of adjunctive therapies for KD across Latin America. IVIG continues to be the initial and resistance treatment, however, steroids are also used and to a lesser extent, biological therapy such as infliximab. Future studies should address the barriers to therapy in children with acute KD throughout Latin America.

## Introduction

Kawasaki disease (KD) is an acute systemic vasculitis in children. Recent studies have implicated inflammatory cytokines and abnormalities in immune regulation as part of the pathophysiology in KD, creating new targets for adjunctive therapy (1–4). Publications about the epidemiology and available drugs and treatment schedules for children with acute KD in Latin America are scarce. One of the largest previous case summaries of children with KD in Mexico reported only 250 children in 32 years (5). In a study of cases in Central America (Guatemala, El Salvador, Honduras, Nicaragua, Costa Rica, and Panama) Ulloa-Gutierrez et al. found that from 2000 to 2010 there were only 11 reports from four countries, mostly consisting of single case reports and small series (6). Of all countries, Costa Rica contributed the highest number of cases (124 cases over 13 years). However, no cases were reported for Nicaragua or Guatemala. (6).

The Latin American KD Network (Red de la Enfermedad de Kawasaki en America Latina, REKAMLATINA) is a standardized registry where data from patients with acute KD throughout Latin American countries has been retrospectively and prospectively collected since January 2009. The network gathers information on demographics, clinical characteristics, laboratory evaluation, response to treatments, and outcomes. The initial and standard treatment for KD has been the use of high dose IV immunoglobulin (IVIG) in combination with aspirin (7). However, in some parts of Latin America, IVIG is not available for first line treatment of KD, and even when available, it may be difficult to acquire or administer early.

Although rates of IVIG resistance are unknown in Latin America, 10–20% of KD patients throughout the United States, Europe, and Asia have IVIG-resistant KD, increasing the risk of developing coronary artery abnormalities (CAA) (8). This population, in particular, may benefit from adjunctive therapy, as recently recommended in the revised 2017 American Heart Association KD guidelines (9). In addition, certain populations like infants with KD have a higher risk of developing CAA and thus may warrant additional adjunctive therapy (10, 11). A number of therapies exist for treating IVIG-resistant KD and those at high risk of CAA, including the second dose of IVIG, steroids, infliximab, or other immunomodulatory therapies (12–14). However, treatment options are limited in some regions of the world, including Latin America. Furthermore, as no study has determined which treatment is best for treating IVIG resistant KD or high-risk KD patients, the treatment choice is left to the treating physician. This study aimed to report the adjunctive therapies used to treat IVIG-resistant and high risk KD patients in Latin America.

## Materials and Methods

### Subjects and Clinical Data

This analysis considers data from patients with KD in the REKAMLATINA registry between January 1, 2009, and May 31, 2017. This analysis included data from the first two multicenter studies of the network, REKAMLATINA-1, and REKAMLATINA-2. The latter study was retrospective and included patients who had a discharge diagnosis of classic or incomplete KD (according to the 2004's AHA diagnostic criteria), admitted from January 1, 2009, to December 31, 2013, at each of the participant referral pediatric or general hospitals. The REKAMLATINA-1 study enrolled KD patients prospectively, who were admitted at any of the participant centers from June 1, 2014, to May 31, 2017. Each patient was treated and followed according to the standard protocol in the referral hospital. The data collected included age at onset, gender, clinical criteria, administered medications, response to therapy, laboratory results, echocardiographic findings, and clinical outcomes.

The internal dimension of the coronary arteries was converted to Z-scores (standard deviations from the mean normalized for body surface area) (Dimensions as published by the American Heart Association). The maximal Z score (Zmax) of the left anterior descending artery (LAD) or right coronary artery (RCA) in the first 8 weeks was calculated.

Laboratories included white blood cell count (WBC), platelet count, erythrocyte sedimentation rate (ESR), C-reactive protein (CRP), albumin, alanine aminotransferase (ALT), gamma glutamyl transpeptidase (GGT), and hemoglobin concentration normalized for age (zHgb). IVIG resistance was defined as fever (≥38 C) more than 36 h, but not more than 7 days after completion of the IVIG without another cause. In assessing IVIG resistance, we included only patients who received IVIG within the first 10 days of fever onset. Patients were classified into 2 groups for analysis: (1) a single dose of IVIG or (2) other therapies, which included those treated with a combination of IVIG and adjunctive therapies, or steroids only.

### Statistical Methods

Continuous variables were summarized as median [interquartile range (IQR), 25th−75th percentile].

Categorical variables were summarized as frequencies and percentages. Demographic, clinical laboratory data and disease outcome among the two groups were compared using the Wilcoxon.

#### Mann-Whitney Test

All statistical analyses were conducted in Graph Pad Prism version 8.1.2 (available at: https://www.graphpad.com).

### Ethics

The study received Institutional Review Board approval at the University of California San Diego as well, as at each institution enrolling subjects in the REKAMLATINA database.

## Results

**Figure 1:**
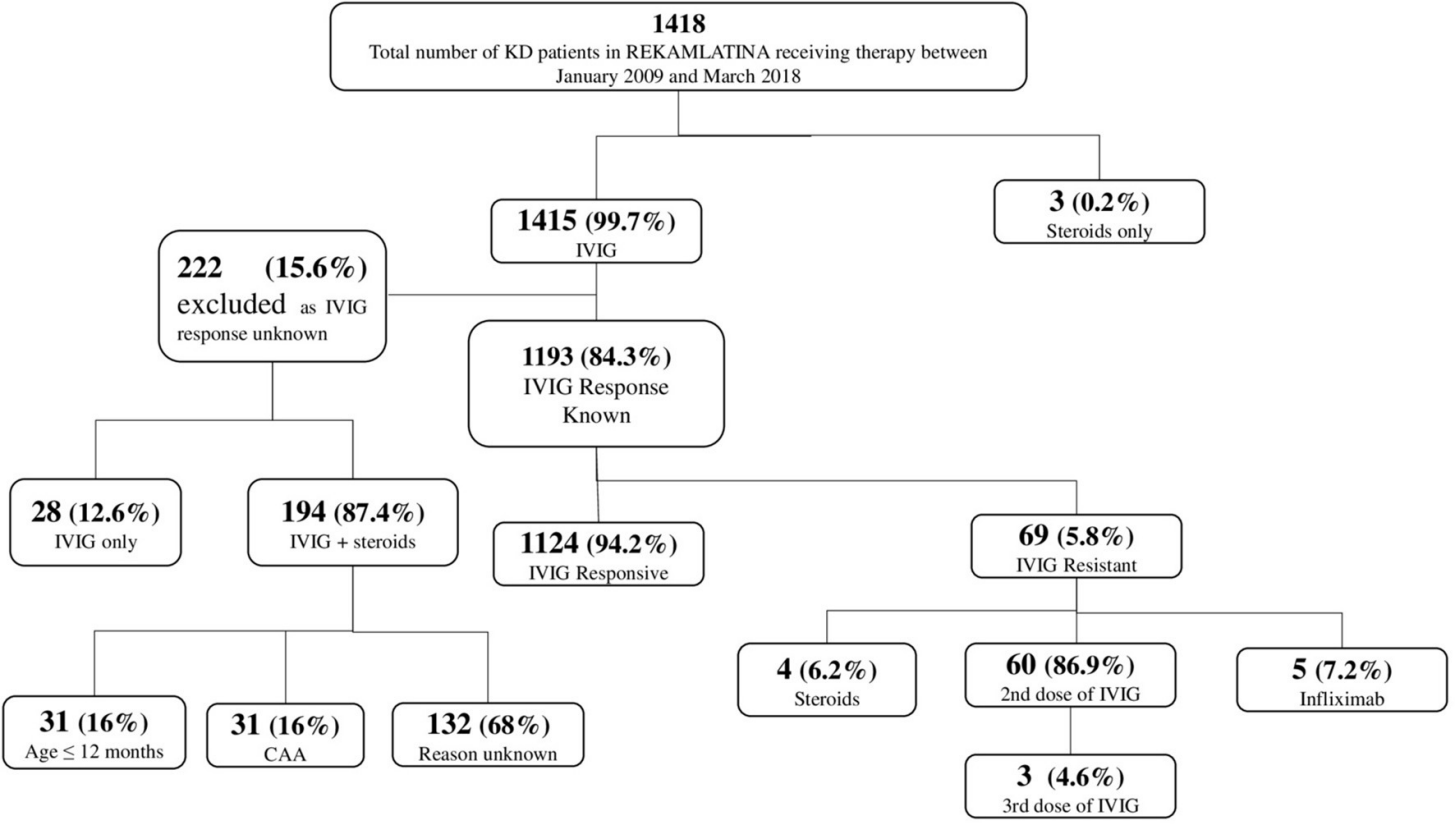
Initial and subsequent treatments administered to 1,418 patients with acute Kawasaki disease in Latin America.

A total of 1,855 patients diagnosed with KD in 18 Latin American countries were included in the Registry (Table 1), of which 437 patients were excluded due to a significant lack of demographic, laboratory, treatment, or echocardiographic data. Of the remaining 1,418 patients, 1,152 received only IVIG (81.2%), and 266 (18.8%) either IVIG in combination with adjunctive therapy or steroids alone (Figure 1). Age at onset was similar in both groups (median 2 vs. 2.2 years, respectively), as was the average days of the patients' illness at the time of hospitalization (median 7.2 vs. 7.3 days). The majority of patients were male (58 vs. 63.9%) and had been hospitalized in the first 10 days of illness (85.1 vs. 84.2%) (Table 2). There were no significant differences in the number of leukocytes, hemoglobin concentration adjusted for age, erythrocyte sedimentation rate, C-reactive protein levels, alanine aminotransferase, or gammaglutamyl transpeptidase at the time of diagnosis between the two groups. When compared to KD patients treated with a single dose of IVIG, those treated with adjunctive therapies had a lower platelet count (423 vs. 375, *P* = < 0.0001) and lower albumin levels (3.3 vs. 3.1, *P* = < 0.0001) (Table 2).

**Table 1:** Number of patients divided by each country (1,418 total patients).

**Country**	**Number of patients contributed**
Argentina	35
Brazil	31
Chile	82
Colombia	146
Costa Rica	215
Cuba	8
Dominican Republic	21
Ecuador	56
Guatemala	25
Honduras	27
Mexico	424
Panama	153
Paraguay	9
Peru	47
Puerto Rico	14
Salvador	92
Uruguay	31
Venezuela	2

**Table 2:** Comparison of demographic, clinical characteristics, and outcome among 1,418 patients with Kawasaki disease in Latin America stratified by treatment.

	**IVIG alone (*n* = 1,152)**	**IVIG and adjunctive therapy (A) (*n* = 266)**	*P*-value^*^
Age in yrs at onset	2 (1.2–3.8)	2.2 (1–4.4)	0.5029
Sex, Male	58%	63.9%	0.0810
Illness day at diagnosis	7.2 (5–8)	7.3(5–9)	0.75
**Illness, day at hospitalization**			
≤ 10 d	85.1%	84.2%	0.1799
>10 d	13%	15.7%	0.2765
White blood cell count K × 103	14.2 (11–18.14)	14.5 (10.55–19.02)	0.8866
zHgb	−1.2 (−2.38 to 0.14)	−0.94 (−2.16 to 0.51)	0.0639
Platelet count K × 10^3^	423 (298–516)	375 (262–453)	<0.0001
ESR mm/h	45 (30–56)	47.5 (28–54)	0.6247
CRP mg/dL	6.7 (2.47–12.2)	7 (2.3–15.8)	0.4800
ALT IU/L	41 (22–87.5)	42 (24–101.8)	0.3716
GGT IU/L	48.5 (20–137.3)	77 (26.2–176)	0.0644
Albumin mg/dL	3.3 (2.9–3.7)	3.1 (2.7–3.4)	<0.0001
Overall coronary artery outcome (Zmax)	0.8 (−0.09 to 2.05)	1.5 (0.57–4.3)	<0.0001
Coronary artery outcome if diagnosed ≤ 10 days (Zmax)	0.73 (−0.14 to 1.84)	5.07 (1.82–11.22)	<0.0001

*ALT, Alanine aminotransferase; CRP, C-reactive protein; ESR, erythrocyte sedimentation rate; GGT, gamma glutamyl transpeptidase; zHgb, hemoglobin concentration normalized for age. Values are median (IQR) unless otherwise noted*.

*(A) Patients were treated with the following treatment combinations: two doses of IVIG, three doses of IVIG, steroids alone, steroids and IVIG, and infliximab*.

* *P-value Wilcoxon-Mann-Whitney test for continuous variables to compare the overall difference among the 2 groups*.

*Data were available for the following number of patients in each variable (IVIG only; IVIG + adjunctive therapy): Age (n = 1,152; n = 266); Sex (n = 669; N = 170); Illness, day at hospitalization ≤ 10 d (n = 981; 319 n = 224) and >10 d (n = 150; n = 42); White blood cell count K × 10 3 (n = 1,130; n = 261); zHgb (n = 1,127; n = 320; 262); Platelet count K × 103 (n = 1,113; n = 260); ESR (n = 715; n = 236); CRP (n = 1,022; n = 219); ALT (n = 969; n = 252); GGT (n = 316; n = 108); albumin (n = 773; n = 219); overall coronary artery outcome (n = 590; n = 86); coronary artery outcome among diagnosed ≤ 10 days (n = 502; n = 20)*.

Of the patients included in this study, 1,415 (99.7%) were treated with IVIG, and 3 (0.2%) patients received only steroids (Figure 1). In total, 263 (18.6%) KD patients received more than a single medication for treatment of KD (*N* = 198, steroids; *N* = 60, additional IVIG; *N* = 5, infliximab). In 1,193 (84.3%) patients, the response to IVIG was reported. Of these, 69 (5.8%) KD patients were noted to be IVIG-resistant and the majority (*N* = 60, 87%) were treated with two doses of IVIG, with three of those patients getting a third dose of IVIG. In the remaining cases, patients were treated with either steroids (*N* = 4, 6.2%) or infliximab (*N* = 5, 7.2%) as rescue therapy. The IVIG response was unknown in 222 patients, 194 of whom received adjunctive therapy with steroids. Of these, 31 (16%) were <12 months old and 31 (16%) had CAA at the time of admission, which could have possibly led to the use of steroids. The rationale for giving only steroids without IVIG to three acute KD patients, specifically the availability of IVIG, is unknown.

With regards to the coronary artery, the Zmax of the coronary arteries was higher in KD patients treated with adjunctive therapy, especially those treated in the first 10 days of illness (0.73 vs. 5.07, *P* < 0.0001, Table 2).

## Discussion

This is the first multinational, multicenter study to report on the use of adjunctive therapies in children with KD from Latin America. While the majority of KD patients only received a single dose of IVIG, nearly 20% of patients were treated with adjunctive therapy after receiving an initial IVIG dose (8). This is consistent with what has been reported in the United States and Japan (15). Although the rationale for using adjunctive therapy was unknown in all these patients, the reasons for some adjunctive treatments were similar to those in other parts of the world, including IVIG-resistance, young age, and CAA. While steroids were the most reported adjunctive therapy, additional doses of IVIG were also administered relatively frequently. This is an interesting issue, given the worldwide shortage of IVIG and the reported risk of hemolytic anemia created by increasing doses of IVIG (16). In this study, we did not have sufficient data to assess whether the anemia seen in some KD patients was hemolytic and whether increasing doses of IVIG was associated with a higher risk of hemolytic anemia. However, a comparative effectiveness study is currently being conducted, examining the use of a second dose of IVIG vs. infliximab in IVIG-resistant KD patients, further evaluating the risk of hemolytic anemia in KD patients (16).

In this analysis, we had a low rate of IVIG-resistance, with only 5.8% of the total patients reported as such. This is most likely due to an underestimation of IVIG-resistance in Latin America, as the worldwide rate ranges from 9 to 20% (15, 17). In previous reports from Latin America, IVIG resistance was reported to be 9% (18) and in multiethnic studies from the United States, IVIG-resistance in Hispanic KD patients was found to be 15% (19). If we were to assume that 132 KD patients received adjunctive treatment with steroids because they had IVIG resistance, the IVIG-resistance rate would be 14.2% (201/1,415).

In this study, 14% of KD patients were treated with steroids as adjunctive therapy, a treatment that is commonly administered worldwide in KD patients. Dominguez et al. found that 7.7% of KD patients were treated with corticosteroids for resistance after a first dose of IVIG at the Children's Hospital of Colorado in the United States (20). Chen et al. reported 37.4% of Chinese KD patients were treated with steroids for resistance to the first dose of IVIG (21). In the most recent epidemiological survey of KD in Japan, 13% of KD patients were reported to have received steroids with initial IVIG given a high likelihood of IVIG-resistance (15).

In our study, only five KD patients were treated with infliximab in addition to IVIG. It may be that infliximab was not available in many centers or that there was limited experience in using the drug, thus, it was not the adjunctive therapy of choice. By comparison, the use of adjunctive infliximab therapy has ranged from 1.4% in Spain to 6.5% in the United States (20). In this study, there were also three patients, all of whom were diagnosed in the first 10 days of illness, who were treated only with steroids (methylprednisolone at 30 mg/kg/day). Although the reason these patients did not receive IVIG is unknown, it is difficult to acquire this drug in some regions of Latin America. This is either because it is not readily or quickly available, or because the cost is not covered by insurance, and might require the patient to pay for it themselves, which may not be feasible.

A lower albumin and platelet count were found in patients treated with adjunctive therapy. It has been reported that a low albumin and platelet count increases the risk for CAA, resistance, or recurrence in KD patients from other regions (9). The later median days of illness at the time of diagnosis in this cohort as compared to other countries may explain these lower albumin levels (22, 23). These patients were likely sicker, which warranted a higher rate of adjunctive therapies. Additionally, it is understandable that patients receiving adjunctive therapy had a higher Zmax, in line with the revised 2017 AHA KD guidelines, which recommend that high-risk KD patients receive more than a single dose of IVIG (9).

As this study is the largest data set to be analyzed for adjunctive treatment in the care of patients with acute KD in Latin America, it has both strengths and limitations. We analyzed KD patients who attended the main pediatric or referral countries in the region, which encompasses 18 countries. The previously mentioned lack of some data which resulted in the exclusion of patients, and the absence of consensus as to when or how to use adjunctive therapies are two limitations. There was also wide variability in reporting about patient responses to IVIG therapy, which limited our ability to calculate the exact IVIG-resistance rate. Furthermore, the lack of long-term follow-up data on patients meant that we were unable to study the impact of adjunctive therapies.

## Conclusion

This is the first report from the Latin American KD Registry, focusing on the use of adjunctive therapies in treating patients with acute KD. This study indicates that a better understanding of treatment trends in Latin America could help improve the standard of care for KD patients in this region.

## Data Availability Statement

The raw data supporting the conclusions of this article will be made available by the authors, without undue reservation, to any qualified researcher.

## Ethics Statement

The study received Institutional Review Board approval at the University of California, San Diego as well as at each individual institution enrolling subjects in the REKAMLATINA database.

## Author Contributions

BF-R, AT, EB, and RU-G were responsible for data analysis and writing of the manuscript. The rest of the co-authors (principal investigators and coinvestigators) revised the manuscript and made intellectual contributions to its contents.

## The REKAMLATINA 1-2 Study Group Investigators

Luisa B. Gámez-González (Hospital Infantil de Chihuahua, Chihuahua, México), Paola Pérez Camacho, Jaime Patiño, Daniela Cleves (Fundación Valle del Lili, Cali, Colombia), Lorena Franco, Nora Bueno, Ana Rosalía Báez (Hospital Infantil Municipal de Córdoba, Córdoba, Argentina), Maria L. Avila-Agüero, Kattia Camacho-Badilla, Alejandra Soriano-Fallas, Mariella Vargas-Gutierrez, Susan Li-Chan, Kathia Valverde (Hospital Nacional de Niños “Dr. Carlos Sáenz Herrera, Centro de Ciencias Médicas de la Caja Costarricense de Seguro Social, San José, Costa Rica), Adrián Collia, Alejandro Ellis (Sanatorio Mater Dei, Buenos Aires, Argentina), Carlos F. Grazioso, Pablo J. Grazioso, Gonzalo Calvimontes (Sanatorio Nuestra Sra. Del Pilar/Hospital General San Juan de Dios, Ciudad Guatemala, Guatemala), Giannina Izquierdo, Pilar Picart, Cecilia Piñera (Hospital de Niños “Dr. Exequiel González Cortés, Santiago, Chile), Marco T. Luque (Hospital Escuela Universitario, Tegucigalpa, Honduras), Mario Melgar (Hospital Roosevelt, Ciudad Guatemala, Guatemala), Andrea Salgado, Arturo Borzutzky, Alfonso Hernández-Ojeda, Pamela Morales-Matamala Pontificia Universidad Católica de Chile, Santiago, Chile. Antonio Arbo, Dolores Lovera Sara Amarilla, Fernando Galeano, Norma Astigarraga (Instituto de Medicina Tropical, Asunción, Paraguay), Maria del Carmen Luis-Álvarez (Hospital Pediátrico Universitario “William Soler”, La Habana, Cuba), Stella Gutierrez, Estefanía Fynn, Elizabeth Assandri, María Caggiani (Hospital CASMU, Montevideo, Uruguay), Jacqueline Levy, Elizabeth Castaño, Raúl Esquivel, Ximena Norero, Scarlet Sinisterra (Hospital del Niño Dr. José Renán Esquivel, Ciudad Panamá, Panamá), Carlos Daza, Carmen Requena (Hospital Materno Infantil José Domingo de Obaldía, Chiriquí, Panamá), Isabel C. Hurtado-Palacios, Antonio Madrid (Hospital Universitario del Valle, Centro Médico Imbanaco, Cali, Colombia), Angélica Calvache-Burbano, Antonio Fernández, Nelly Chávez-Solórzano, Marianella Layana-Coronel, Denisse Olaya-González, Yasmín Sánchez, Dolores Freire (Hospital del Niño “Dr. Francisco de Icaza Bustamante, Guayaquil, Ecuador), Marco A. Yamazaki-Nakashimada, Raymundo Rodríguez-Herrera (Instituto Nacional de Pediatría, Ciudad de México, México), Sarbelio Moreno-Espinosa, Ángel Flores, Ana V. Villarreal (Hospital Infantil de México Federico Gómez, Ciudad de México, México), Diana López-Gallegos, Horacio Márquez-González (Hospital Infantil Privado, Ciudad de México, México), Adriana Díaz-Maldonado, Kelly Marquez-Herrera, Roy Sanguino-Lobo (Fundación HOMI Hospital Pediátrico de la Misericordia; Bogotá, Colombia), Natalia Lara (Universidad Nacional de Colombia & Fundación HOMI Hospital Pediátrico de la Misericordia, Bogotá, Colombia), Neusa Keico Sakita, María Fernanda Pereira Badue, Gabriela Leal (Hospital Das Clinicas da Faculdade Medicina da USP, São Paolo, Brazil), Diana C. Medina, María Fernanda García-Venegas, Pilar Guarnizo, Manuel Huertas-Quiñones, Paula A. Araque, Claudia Stapper (Fundación Cardioinfantil & Universidad El Bosque, Bogotá, Colombia), Pio López (Hospital Universitario del Valle, Cali, Colombia), Jaime Deseda-Tous, Margarita Martínez-Cruzado, Rubén Díaz Rodríguez (Hospital Español Auxilio Mutuo, San Juan, Puerto Rico), Mónica Pujadas, Karina Machado, Federica Badía, Alejandra Vomero (Hospital Pediátrico Centro Hospitalario Pereira Rossell, Montevideo, Uruguay), Jorge A. Vázquez-Narváez, Norma J. Cortés-Cruz (Hospital Infantil “Eva Sámano de López Mateos”, Morelia, México), Mussaret B Zaidi (Hospital General Dr. Agustín O'Horán, Yucatán, México), Mildred Zambrano, Joyce Andrade, Juan Chang-Asinc (Hospital de Niños Dr. Roberto Gilbert Elizalde, Guayaquil, Ecuador), Guillermo Barahona, Mauricio Velado, Mario Gamero (Hospital de Niños Benjamín Bloom; San Salvador, El Salvador), Guillermo Soza, Carolina Cerda (Hospital Dr. Hernán Enríquez Aravena, Temuco, Chile), Alejandra Sandoval-Carmona (Hospital Dr. Sotero del Río, Santiago, Chile), Guadalupe Urrea (Hospital General de Tijuana, México), Josué Rodríguez-Ríos (Hospital de Especialidades Instituto Hondureño de Seguridad Social, Tegucigalpa, Honduras), María Camila Reyes (Clínica Colsanitas, Bogotá, Colombia), Yokaira Ferreira (Hospital Infantil “Dr. Robert Reid Cabral”, Santo Domingo, Dominican Republic), Rafael Hernández-Magaña, Ignacio Camacho-Meza, Eunice Sandoval-Ramírez, Rubén Alba-Medina (Hospital de Especialidades Pediátrico de León, Guanajuato, México), Julieta González-Palacios (Centenario Hospital Miguel Hidalgo; Aguas Calientes, México), Enrique López-Valentín, Norma D. López-Lara (Hospital para el Niño de Toluca, Toluca, México), Tibisay Triana (Hospital Universitario “Luis Razetti”, Barcelona, Venezuela), Jesús Alvelo (Puerto Rico Children's Hospital, San Juan, Puerto Rico), Sergio Bernal-Granillo (Hospital General de Zona 1/IMMS/Hospital Ángeles CMP, San Luis Potosí, México), Saulo Duarte Passos (Hospital Universitario da Faculdade de Medicina de Jundiai, São Paulo, Brasil), Nadina Rubio-Pérez, Fernando García-Rodríguez (Hospital Universitario, Universidad Autónoma de Nuevo León, Monterrey, México), Rogelio Martínez-Ramírez, Lorena Rodríguez-Muñoz, Karina Flores Hernández (Hospital del Niño “Federico Gómez Santos”, Saltillo, México), Víctor H. Velazco, Patricio Andrade (Hospital del Niño “Dr. Ovidio Aliaga Uria”, La Paz, Bolivia), Alejandro Díaz-Díaz, Juan G Mesa-Monsalve (Hospital General de Medellín, Colombia), Iván F. Gutiérrez-Tobar (Clínica Infantil Colsubsidio, Bogotá, Colombia), Rocío A. Peña-Juárez, Gabriel Vega-Cornejo (Hospital General de Occidente, Jalisco, México), María Mercedes Somarriba, Mariangeles Pérez (Hospital Infantil Manuel de Jesús Rivera, Managua, Nicaragua), Belén Amorín (Hospital Escuela del Litoral Paysandú, Paysandú, Uruguay), Jesús de Lara-Huerta (Hospital Infantil Universitario de Torreón, México), Ana M González-Ortiz (Hospital del Niño y la Mujer “Dr. Alberto Lópe Hermosa”, San Luis Potosí, México), Pablo García-Munitis (Hospital El Cruce, Buenos Aires, Argentina), Alessandra Geisler Lopes, Aline da Graça Fevereiro, Carlota Mott (Hospital Infantil Menino Jesus, São Paulo, Brasil).

## Conflict of Interest

The authors declare that the research was conducted in the absence of any commercial or financial relationships that could be construed as a potential conflict of interest.
